# Association of serum occludin levels and perihematomal edema volumes in intracranial hemorrhage patients

**DOI:** 10.1111/cns.14450

**Published:** 2023-09-18

**Authors:** Shuhua Yuan, Qingfeng Ma, Chengbei Hou, Yue Zhao, Ke Jian Liu, Xunming Ji, Zhifeng Qi

**Affiliations:** ^1^ Cerebrovascular Diseases Research Institute Xuanwu Hospital of Capital Medical University Beijing China; ^2^ Department of Neurology Xuanwu Hospital of Capital Medical University Beijing China; ^3^ Center for Evidence‐Based Medicine, Xuanwu Hospital Capital Medical University Beijing China; ^4^ Clinical Lab, Xuanwu Hospital Capital Medical University Beijing China; ^5^ Department of Pathology, Renaissance School of Medicine Stony Brook University Stony Brook New York USA; ^6^ Center of Stroke, Beijing Institute for Brain Disorders Capital Medical University Beijing China

**Keywords:** biomarker, blood–brain barrier, correlation, intracerebral hemorrhage, perihematomal edema volumes, serum occludin levels

## Abstract

**Background and Purpose:**

Perihematomal edema (PHE) is one of the severe secondary damages following intracranial hemorrhage (ICH). Studies showed that blood–brain barrier (BBB) injury contributes to the development of PHE. Previous studies showed that occludin protein is a potential biomarker of BBB injury. In the present study, we investigated whether the levels of serum occludin on admission are associated with PHE volumes in ICH patients.

**Methods:**

This cross‐sectional study included 90ICH patients and 32 healthy controls.The volumes of hematoma and PHE were assessed using non‐contrast cranial CT within 30 min of admission. Blood samples were drawn on admission, and the levels of baseline serum occludin were detected using enzyme‐linked immunosorbent assay. Partial correlation analysis and multiple linear regression analysis were performed to evaluate the association between serum occludin levels and PHE volumes in ICH patients.

**Results:**

The serum occludin levels in ICH patients were much higher than health controls (median 0.27 vs. 0.13 ng/mL, *p* < 0.001). At admission, 34 ICH patients (37.78%) had experienced a severe PHE (≥30 mL), and their serum occludin levels were higher compared to those with mild PHE (<30 mL) (0.78 vs. 0.21 ng/mL, *p* < 0.001). The area under the receiver operating characteristics curve (ROC) of serum occludin level in predicting severe PHE was 0.747 (95% confidence interval CI 0.644–0.832, *p* < 0.001). There was a significant positive correlation between serum occludin levels and PHE volumes (partial correlation *r* = 0.675, *p* < 0.001). Multiple linear regression analysis showed that serum occludin levels remained independently associated with the PHE volumes after adjusting other confounding factors.

**Conclusion:**

The present study showed that serum occludin levels at admission were independently correlated with PHE volumes in ICH patients, which may provide a biomarker indicating PHE volume change.

## Graphical abstract

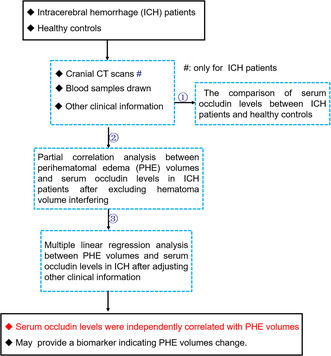

## 1 INTRODUCTION

Intracerebral hemorrhage (ICH) is a severe type of stroke, accounting for 27.9% (about 3.41 million patients) of all prevalent stroke cases worldwide.^1^ Studies have shown that about half of the patients had poor prognoses at 30 days after the occurrence of ICH.^2^, ^3^

Perihematomal edema (PHE), characterized as hypodensity areas surrounding the hematoma, on computed tomography (CT) scans, is one of the severe secondary damages following ICH.^4^ Several clinical studies have reported that the PHE volume was rapidly developed within the first 24 h post‐ICH onset, which was associated with early neurological deterioration following ICH, resulting in poor prognoses.^5^, ^6^, ^7^ Therefore, it is important to be able to frequently and conveniently evaluate the levels of PHE in ICH patients within 24 h after symptom onset, which may assist neurologists in making decisions regarding diagnoses and treatment strategies in a timely manner. As CT or magnetic resonance imaging (MRI) scanning is time‐consuming or cannot be frequently used, it is important to identify blood biomarkers that indicate the PHE volume.

Recent studies have shown that blood–brain barrier (BBB) damage was highly associated with PHE development.^8^, ^9^, ^10^ In addition, experimental studies also reported that ICH induced thrombin formation, inflammation, and erythrocyte lysis in the surrounding parenchyma, resulting in BBB disruption, and in accelerating development of PHE during the acute phase (4–72 h) of ICH.^11^, ^12^

Occludin has been identified as a key protein of tight junction structures and contributes to BBB integrity.^13^ Our previous animal studies showed that occludin in microvessels was degraded during cerebral ischemia, with fragments of occludin entering the blood circulation.^14^ Moreover, the level of occludin fragments in serum was correlated with BBB permeability, suggesting that serum occludin levels could reflect the degree of BBB injury after ischemic stroke.

In the present study, we determined whether serum occludin levels on admission reflected PHE volumes in ICH patients, which could provide potential clinical biomarkers for PHE after ICH.

## 2 METHODS

### 2.1 Participants and study design

This cross‐sectional study was approved by the Ethics Committee of Xuanwu Hospital of Capital Medical University. Written informed consents were obtained before enrollment.

The enrolled cases were consecutively screened from 135 ICH patients who were admitted to the Department of Emergency Neurology of Xuanwu Hospital of Capital Medical University between February 2021 and May 2021. Inclusion criteria were as follows: (1) age ≥ 18 years, (2) ICH observed on initial CT scans within 30 min of admission, (3) time from onset to hospital <24 h, (4) blood samples collected within 30 min of admission, and (5) informed consents were obtained. Exclusion criteria included the following: (1) intracerebral hemorrhage associated with trauma, aneurysm, or arteriovenous malformation; (2) brain tumor stroke, hemorrhagic cerebral infarction, isolated ventricular hemorrhage, or subtentorial intracerebral hemorrhage; (3) inflammatory or infectious diseases, malignant diseases, or immunosuppressive treatment; (4) severe coagulation disturbance disease, severe liver, or renal failure; (5) pregnancy; and (6) blood samples occurred hemolysis or chylemia (turbid or milky white to the naked eyes, triglyceride >4.66 mmol/L).^15^, ^16^

Healthy controls were recruited from the Physical Examination Center of the Xuanwu Hospital of Capital Medical University on May 20, 2021 using questionnaires and physical examinations. Inclusion criteria were the following: (1) age ≥ 18 years, (2) no previous history of central nervous system diseases or psychiatric disorders, (3) blood samples were obtained, and (4) informed consents were obtained. Exclusion criteria included the following: (1) asymptomatic central nervous system diseases discovered by current neurological examinations, (2) inflammatory or infectious diseases, malignant diseases, and immunosuppressive treatment; (3) severe coagulation disturbance disease, severe liver, or renal failure; (4) pregnancy; and (5) blood samples involved hemolysis or chylemia (turbid or milky‐white to the naked eyes, triglyceride >4.66 mmol/L).^15^, ^16^

**FIGURE 1:**
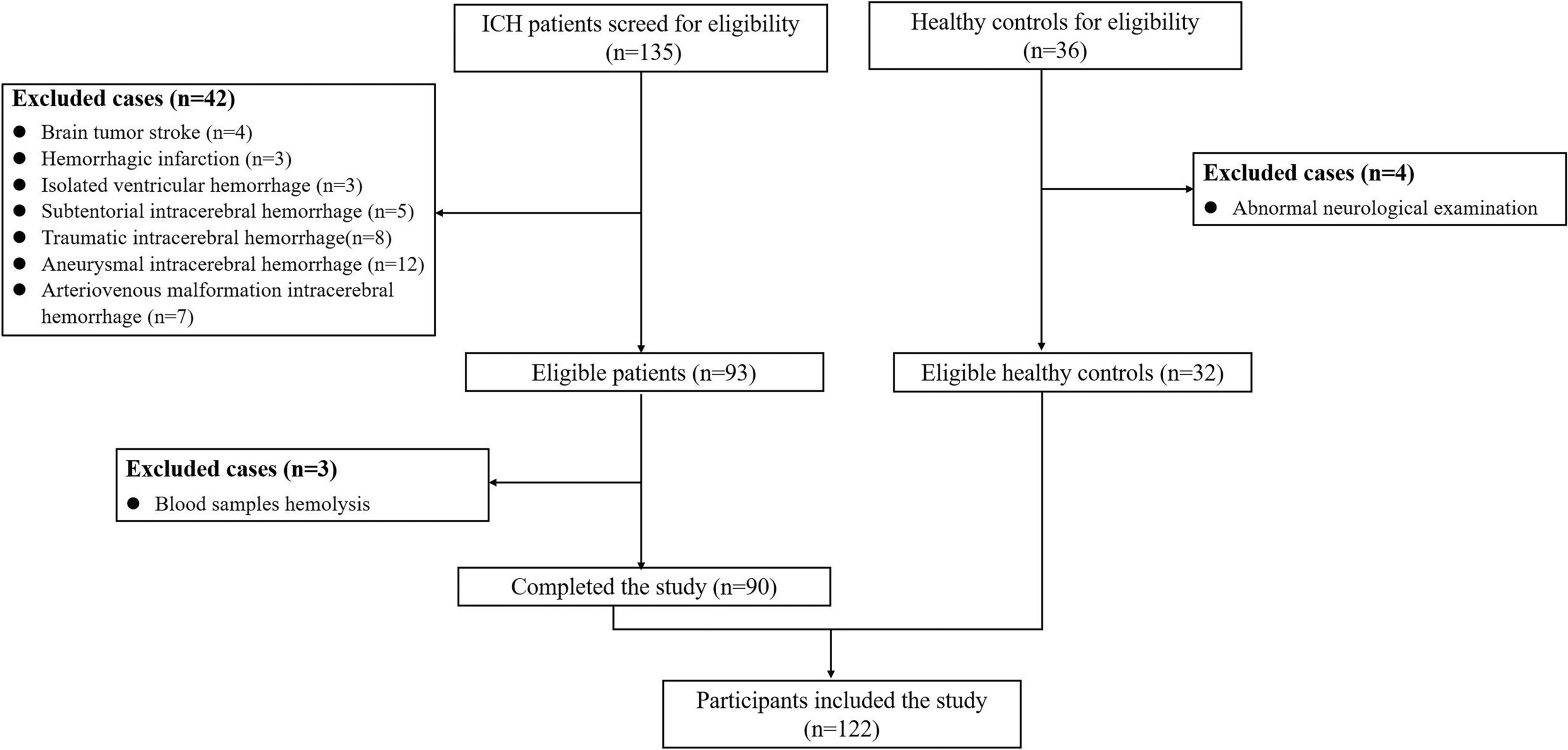
The flow chart of case recruitment. According to inclusion and exclusion criteria, a total of 90 patients with ICH and 32 healthy controls were included in the present study. ICH, intracranial hemorrhage.

In total, 122 participants completed the study, including 90 ICH patients and 32 healthy controls (Figure 1).

### 2.2 Clinical information collection

The baseline clinical information of all cases was collected from questionnaires and electronic medical records (Tables 1 and 2). Stroke severities were assessed by trained neurologists at admission using the National Institutes of Health Stroke Scale (NIHSS), modified Rankin Scale, and Glasgow Coma Scale. The investigators, who collected the clinical data, were blinded to the measurements of blood samples and imaging data.

**TABLE 1:** Comparison of baseline characteristics between ICH patients (*n* = 90) and healthy controls (*n* = 32).

Characteristics	ICH patients	Healthy controls	*p* Value
Male, *n* (%)	62 (68.9%)	16 (50.0%)	0.056
Age (years), mean ± SD	62.09 ± 14.22	61.09 ± 12.78	0.387
Medical history, *n* (%)			
Hypertension	67 (74.4%)	22 (68.8%)	0.619
Diabetes mellitus	22 (24.4%)	13 (40.6%)	0.271
Coronary heart disease	15 (16.7%)	9 (28.1%)	0.273
Hyperlipidemia	25 (27.8%)	13 (40.6%)	0.221
Current smoking	51 (56.7%)	14 (43.8%)	0.251
History of stroke	15 (16.7%)	–	–
History of antithrombotic therapy	11 (12.2%)	4 (12.5%)	0.832

**TABLE 2:** Baseline characteristics of ICH patients on admission within 24 h since stroke onset (*n* = 90).

Characteristics	Values
Male, *n* (%)	62 (68.9%)
Age (years), mean ± SD	62.09 ± 14.22
*Medical history, n (%)*	
Hypertension	67 (74.4%)
Diabetes mellitus	22 (24.4%)
Coronary heart disease	15 (16.7%)
Hyperlipidemia	25 (27.8%)
Current smoking	51 (56.7%)
History of stroke	15 (16.7%)
History of antithrombotic therapy	11 (12.2%)
*Radiologic characteristics on initial CT*	
Location of hemorrhage, *n* (%)	
Isolated ICH	53 (58.9%)
Intraventricular hemorrhage extension	37 (41.1%)
Hematoma volume (mL)	23.88 (9.02–47.96)
Perihematomal edema volume (mL)	22.48 (8.56–42.50)
*Clinical measures*	
ICH score	
≤3 scores	75 (83.3%)
>3 scores	15 (16.7%)
In‐hospital mortality within 24 h	7 (7.8%)
Consciousness (GCS score), *n* (%)	
14–15 (normal)	40 (44.4%)
8–13 (somnolence)	26 (28.9%)
0–7 (coma)	24 (26.7%)
NIHSS score on admission, *n* (%)	
0–4 (mild stroke)	15 (16.7%)
5–15 (moderate stroke)	40 (44.4%)
16–20 (moderate–severe stroke)	7 (7.8%)
21–42 (severe stroke)	28 (31.1%)
Time from onset to blood sampling (min), median (IQR)	188 (116.25–399.25)
Time from onset to head CT scan (min), median (IQR)	198 (119–416)
Baseline SBP (mmHg), mean ± SD	173.19 ± 34.73
Baseline DBP (mmHg), mean ± SD	96.01 ± 19.19
*Laboratory tests at admission*	
WBC (×10^9^/L), mean ± SD	9.91 ± 4.88
Blood glucose (mmol/L), median (IQR)	6.73 (5.70–8.87)
LDL (mmol/L), mean ± SD	2.67 ± 0.83
Plaque count (×10^9^/L), mean ± SD	220.69 ± 66.60
APTT (s), median (IQR)	33.95 (29.66–37.48)
Fibrinogen (g/L), mean ± SD	3.58 ± 0.82
Serum occludin (ng/mL), median (IQR)	0.27 (0.14–1.00)

Abbreviations: APTT, activated partial thromboplastin time; DBP, diastolic blood pressure; GCS, Glasgow Coma Scale; ICH, intracranial hemorrhage; IQR, interquartile range; LDL, low‐density lipoprotein; NIHSS, National Institutes of Health Stroke Scale; SBP, systolic blood pressure; WBC, white blood cells.

### 2.3 PHE volume measurements

Imaging data (hemorrhage location, hematoma volume, and PHE volume) were analyzed by two experienced neuroimaging radiologists. If the evaluation results from the two radiologists greatly differed, a senior radiologist further verified the data. All radiologists were blinded to clinical information and blood data.

**FIGURE 2:**
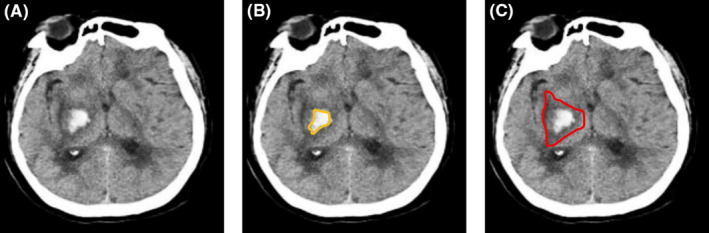
The measurement of PHE volumes on one representative CT image. (A) A representative CT image of ICH at right basal ganglia; (B) The hematoma area (hyperdense lesion, Hounsfield unit 40–100) was manually circled in yellow; (C) The total lesion area [PHE (the area in hypodensity surrounding hematoma, Hounsfield unit 5–33) plus hematoma] was manually outlined in red. The area of PHE on each slice was calculated by subtracting the hematoma area (yellow) from the total lesion area (red). Finally, the PHE volume = ∑ (PHE area in each slice) × the thickness of each slice. ICH, intracranial hemorrhage; PHE, perihematomal edema.

PHE refers to the hypodense region around the hematoma (hyperdense area) on cranial CT scans. The volumes of PHE were semiautomatic analyzedusing archiving communication system and RadiAnt DICOM viewer software (version 2022.1.1).^17^ The total lesion (the area of the hypodense region plus hematoma) and hematoma were outlined and the area of the PHE on each slice was calculated by subtracting the hematoma area from the total lesion area (Figure 2). The volume of the PHE = ∑(PHE area on each slice) × the thickness of each slice.

### 2.4 Measurement of occludin levels in sera

Blood samples were collected into vacuum tubes without anticoagulants within 30 min of admission. Sera were separated at 3000 g at 4°C and stored at −80°C for further use. The levels of serum occludin were measured using a commercial enzyme‐linked immunosorbent assay (ELISA) kit for occludin (Lifespan BioSciences) and strictly followed the instructions and procedures of manual.

### 2.5 Statistical analysis

Statistical analysis was conducted using SPSS statistical software for Windows, version 26.0 (SPSS) and MedCalc software (version 20.10, LTD).Kolmogorov–Smirnov test was used to assess the normality of continuous variables. Categorical variables were recorded as frequencies (%), and the chi‐squared test or Fisher's exact test was used to evaluate the association of categorical variables. Continuous variables for normal distribution were presented as the mean ± standard deviation and compared using the *t*‐test. For non‐normal distribution data, variables were expressed as medians (interquartile ranges, IQRs) and analyzed using Mann–Whitney U test. The statistical significance for all tests was set *p* < 0.05.

The receiver operating characteristics (ROC) curve was used to assess predictive efficacy. ROC‐related indexes were also calculated, including the area under the ROC curve, sensitivity, specificity, positive predictive value, negative predictive value, positive likelihood ratio, and negative likelihood ratio. The best cut‐off value of serum occludin level was obtained using the maximum value of the Youden Index. Pearson's correlation analysis was used to analyze the associations between variables, while partial correlations analysis was used to assess the net correlations of variables. Linear regression models were constructed to analyze the correlations of baseline serum occludin levels (independent response) with PHE (response variable) volumes. To satisfy the requirement of a statistically normal distribution of linear regression and correlation analysis, continuous variables for non‐normal distribution were converted to normal distribution (lg).^18^ The variables with *p <* 0.1 from the simple linear regression analyses were included in the multiple linear regression model.^19^ The variance inflation factor (>10) and tolerance (<0.1) were applied to make sure the variables in the regression analysis were in absence of multicollinearity.^20^, ^21^

The present study is the first to report the association of serum occludin level with PHE.Sample size was calculated, based on our preliminary pilot experiment (means ± SD) and incidence rate of ICH (27.9%) in 2019.^1^ The estimated sample size was 117 cases in total, including 33 healthy controls and 84 ICH patients (32 healthy controls and 90 ICH cases in fact).

## 3 RESULTS

### 3.1 Characteristics and clinical variables of the enrolled cases

A total of 122 cases (90 ICH patients and 32 healthy controls) finally completed the present study, based on the inclusion and exclusion criteria (Figure 1). The baseline clinical variables are listed in Table 1. Statistical analyses showed that there was no significant difference between healthy controls and ICH patients among baseline characteristics, which included age, sex, and previous medical history (hypertension, coronary heart disease, diabetes, hyperlipidemia, and current smoking).

### 3.2 Difference in baseline serum occludin levels between ICH patients and healthy controls

**FIGURE 3:**
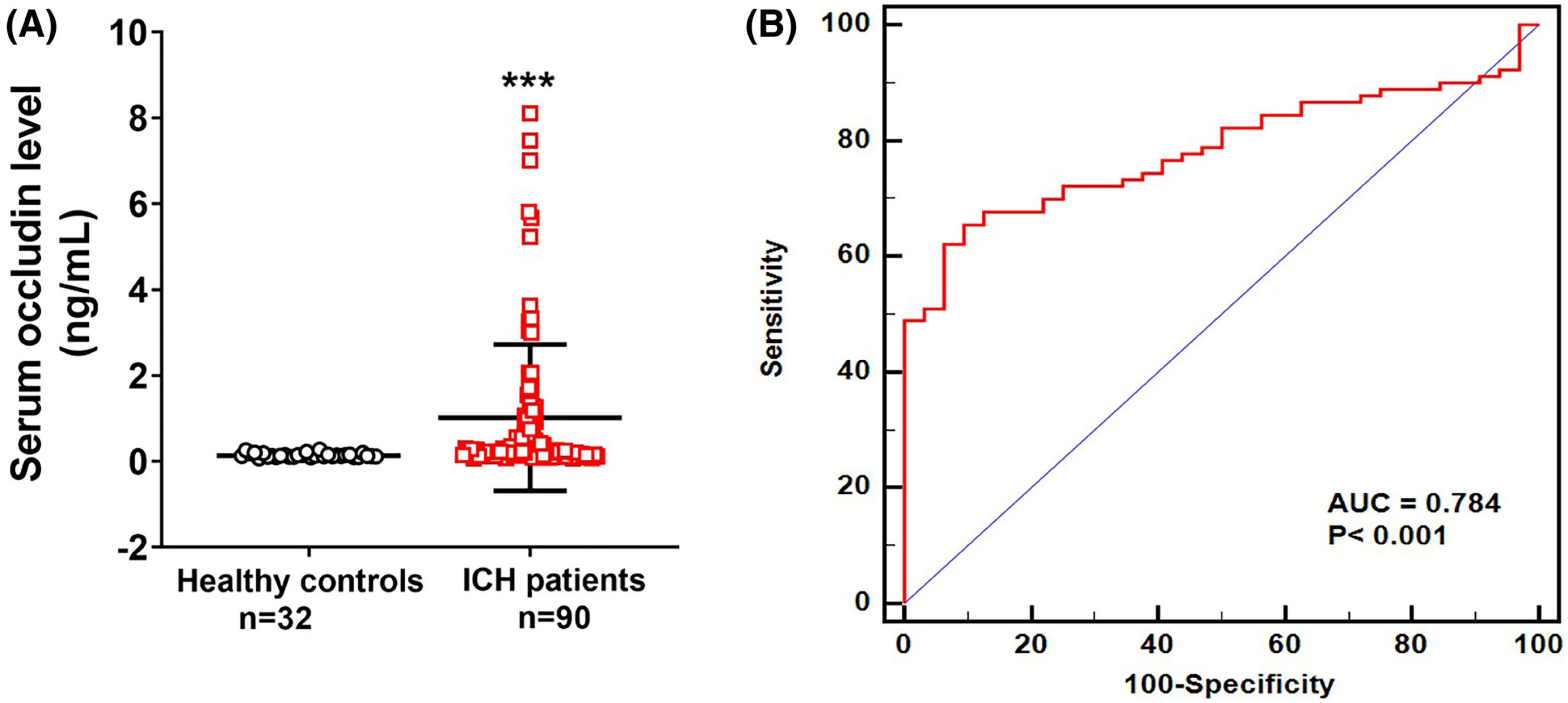
Difference in analysis of serum occludin levels between ICH patients and controls. (A) Compared to healthy controls, the serum occludin levels in patients with ICH were significantly higher (0.27 [IQR, 0.14–1.01] vs. 0.13 [IQR, 0.11–0.16], *p* < 0.001). (B) The area under the ROC curve for distinguishing patients with ICH from healthy controls was 0.784 (95% CI: 0.701–0.854, *p* < 0.001). Data were presented as median (IQR). CI, confidence interval; ICH, intracranial hemorrhage; IQR, interquartile range; ROC, receiver operator characteristic curve.

To determine whether the baseline serum occludin levels differed between ICH and the control groups, serum samples were collected at admission and the occludin levels were measured using ELISAs. The results showed that the baseline levels of serum occludin in ICH patients were significantly higher than those in healthy controls (0.27 [IQR, 0.14–1.01] vs. 0.13 [IQR, 0.11–0.16] ng/mL; *p* < 0.001) (Figure 3A).

The ROC curve was used to determine whether the baseline level of serum occludin could distinguish ICH patients from healthy controls. The area under the ROC curve (AUC) for distinguishing patients with ICH from healthy controls was 0.784 (95% confidence interval [CI]: 0.701–0.854; *p* < 0.001) (Figure 3B). A cut‐off value of 0.202 ng/mL identified ICH patients with a sensitivity of 65.6%, specificity of 90.6%, positive predictive value of 95.2%, negative predictive value of 48.3%, positive likelihood ratio of 6.99, and negative likelihood ratio of 0.38. After adjusting clinical variables (age, sex, family history of hypertension, coronary heart disease, hyperlipidemia, diabetes, and current smoking), a high level of baseline occludin (>0.202 ng/mL) was an important factor, which could distinguish ICH patients from healthy controls (adjusted odds ratio [OR], 22.3; 95% CI, 5.8–86.1; *p* < 0.001).

### 3.3 The baseline level of occludin is associated with the severity of the PHE, but not the hematoma volume

Based on occludin protein as a potential biomarker for cerebral ischemia‐induced BBB injury and BBB disruption after ICH onset being engaged in PHE development, we further focused on ICH patients to determine whether the baseline level of occludin was associated with the severity of the PHE.

Baseline clinical variables are listed in Table 2. Among the ICH patients, isolated ICH patients (*n* = 53) accounted for 58.9%, while ICH patients complicated with intraventricular hemorrhage (*n* = 37) accounted for 41.1%. Based on initial CT scans, the median hematoma volume was 23.88 (IQR, 9.02–47.96) mL, while the median PHE volume was 22.40 (IQR, 8.56–42.50) mL.

**FIGURE 4:**
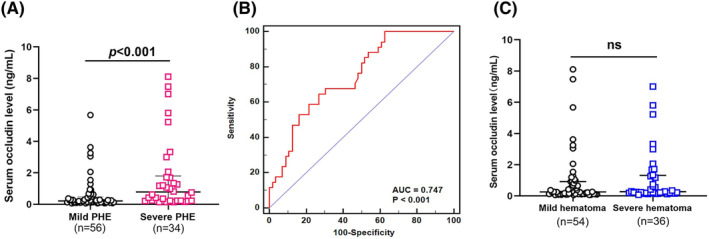
The relationship of serum occludin level and PHE or hematoma severity. (A) The levels of serum occludin in patients with severe PHE were higher than that with mild PHE (0.78 [IQR, 0.23–1.80] vs. 0.21 [IQR, 0.12–0.48] ng/mL; *p* < 0.001). (B) The ROC curve was used to further evaluate the association of serum occludin levels with PHE severities (AUC, 0.747; 95% CI, 0.644–0.832; *p* < 0.001). (C) There was no significant difference in serum occludin levels between the two groups (severe hematoma group: 0.26 [IQR 0.13–0.92] ng/mL vs. mild hematoma group: 0.27 [IQR, 0.20–1.32] ng/mL; *p* = 0.220).Data were presented as median (IQR). AUC, area under the curve; ICH, intracranial hemorrhage; IQR, interquartile range; PHE, perihematomal edema; ROC, receiver operator characteristic curve.

Based on the larger PHE volume (≥30 mL) being associated with poor outcome, ICH patients were divided into two groups: mild PHE (<30 mL, *n* = 34) and severe PHE (≥30 mL, *n* = 56). Statistical analysis showed that the level of serum occludin in patients with severe PHE was higher than that with mild PHE (0.78 [IQR, 0.23–1.80] vs. 0.21 [IQR, 0.12–0.48] ng/mL; *p* < 0.001) (Figure 4A). The ROC curve was used to further evaluate the association of serum occludin levels with PHE severities (AUC, 0.747; 95% CI, 0.644–0.832; *p* < 0.001) (Figure 4B). A cut‐off value of 0.376 ng/mL identified serious PHE patients with a sensitivity of 64.7%, specificity of 73.2%, positive predictive value of 59.5%, negative predictive value of 77.4%, positive likelihood ratio of 2.42, and negative likelihood ratio of 0.48. After adjusting for age, sex, family history of hypertension, coronary heart disease, hyperlipidemia, diabetes, and current smoking, a high level of baseline serum occludin (>0.376 ng/mL) distinguished severe PHE patients from mild PHE (OR, 5.3; 95% CI, 1.9–14.4; *p* = 0.001).

Based on the larger volume of hematoma being related with PHE severity, ICH patients were divided into two groups: severe hematoma (≥30 mL, *n* = 36) and mild hematoma (<30 mL, *n* = 54). Statistical analysis showed that there was no significant difference in serum occludin levels between the two groups (severe hematoma: 0.26 [IQR, 0.13–0.92] vs. mild hematoma 0.27 [IQR, 0.20–1.32] ng/mL; *p* = 0.220; Figure 4C).

These results suggested that serum occludin levels on admission were closely associated with PHE volumes, but not with hematoma volumes.

### 3.4 The baseline level of serum occludin is independently associated with the PHE volumes in ICH patients

Pearson's correlation analysis was conducted to determine whether the baseline level of serum occludin was correlated with the PHE volume. According to the results in Table 3, hematoma volume was correlated with the PHE volume (*r* = 0.926, *p* < 0.001). Moreover, PHE volume (*r* = 0.426, *p* < 0.001) was higher correlated with serum occludin level than hematoma volume (*r* = 0.230, *p* < 0.05). Therefore, in the present study, hematoma volume was considered as a confounding factor for the association of serum occludin levels and PHE volume.

**TABLE 3:** Pearson's correlations of baseline serum occludin level, baseline hematoma volume, and perihematomal edema volume.

*r*	Serum occludin level	Hematoma volume	Perihematomal edema volume
Serum occludin level	–	0.230*	0.462***
Hematoma volume	0.230*	–	0.926***
Perihematomal edema volume	0.462***	0.926***	–

*Note*: The data in table were converted to lg to meet statistical requirements. *p < 0.05, ***p < 0.001.

**FIGURE 5:**
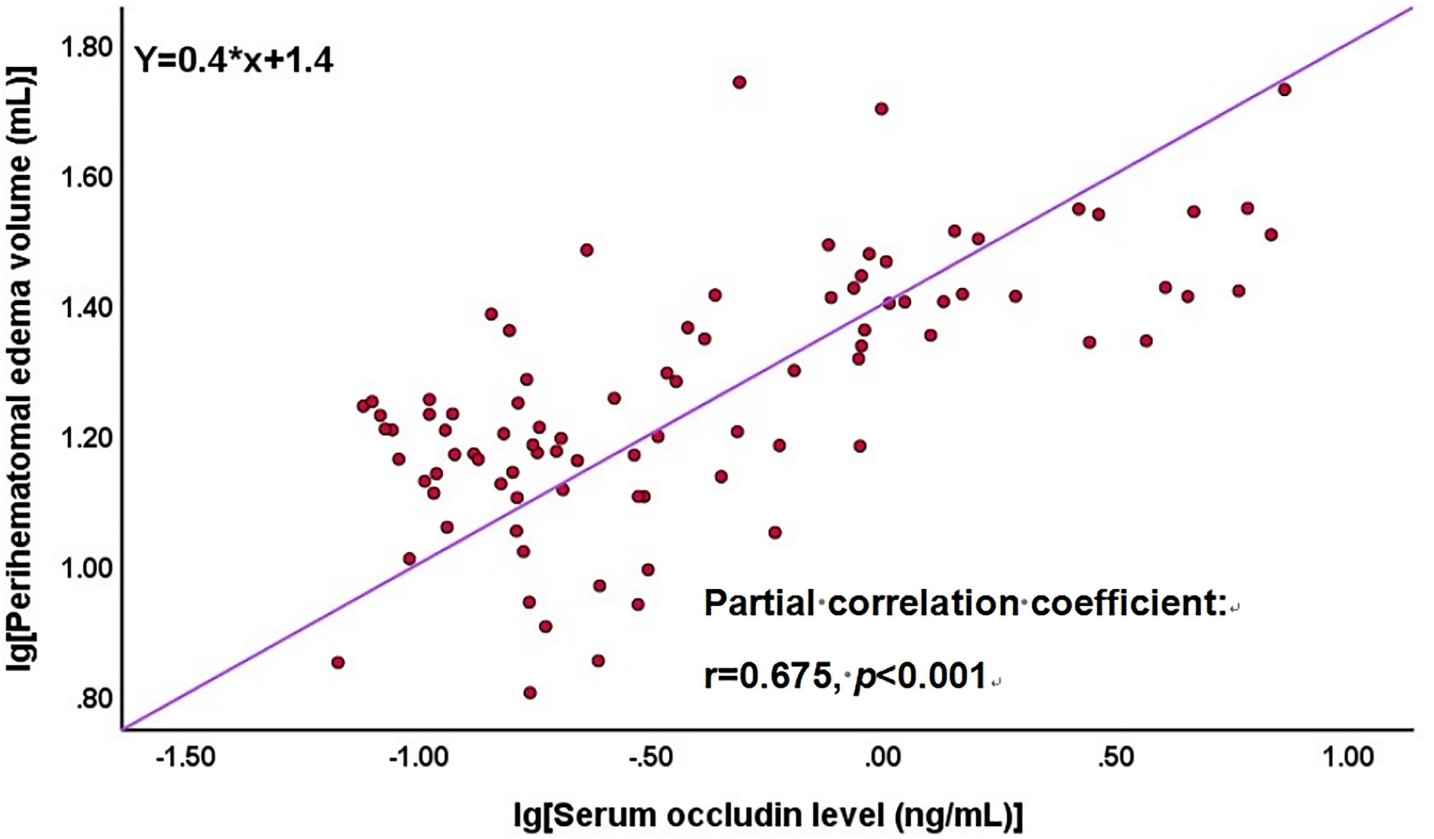
Partial correlation analysis of the serum occludin levels on admission and PHE volumes. To determine the net correlation between baseline serum occludin levels and PHE volumes, partial correlation analysis was performed to exclude the interference of hematoma volumes. Partial correlation analysis showed that a high baseline level of serum occludin showed a linear correlation with the PHE volume (*r* = 0.675; *p* < 0.001), after adjusting hematoma volumes. PHE, perihematomal edema.

To determine the net correlation between baseline serum occludin levels and PHE volumes, partial correlation analysis was performed to exclude the interference of hematoma volumes. Partial correlation analysis showed that a high baseline level of serum occludin showed a linear correlation with the PHE volume (*r* = 0.675; *p* < 0.001; Figure 5), after adjusting hematoma volumes.

To determine whether levels of baseline serum occludin were independently associated with PHE volumes in ICH patients, we conducted simple and multiple linear regression analyses. Firstly, baseline characteristics of ICH patients were screened using simple linear regression, showing that nine candidate variables (*p* < 0.1) being associated with PHE volumes, which included sex, current smoking, Glasgow Coma score, NIHSS score, blood glucose, activated partial prothrombin time, hematoma volumes, white blood cells and the baseline levels of serum occludin. Finally, multiple linear regression analysis showed that the levels of baseline serum occludin remained independently associated with the PHE volumes (*p* < 0.001; Table 4), after adjusting the other eight factors listed above.

**TABLE 4:** Simple and multiple linear regression analysis to evaluate the association of baseline characteristics and PHE volumes (mL) on admission in ICH patients (*n* = 90).

Characteristics	Simple linear regression				Multipleline regression				Multicollinearity	
	β coefficient 95% CI			*p*	β coefficient 95% CI			*p*	Tolerance	VIF
*Demographics*										
Age	0.229	−0.578	1.216	0.646	–	–	–	–	–	–
Sex										
Male	1 (reference)	–	–	–	1 (reference)	–	–	–	–	–
Female	0.238	0.011	0.464	**0.040**	0.068	−0.027	0.164	0.159	0.456	2.194
*Medical history*										
Hypertension										
No	1 (reference)	–	–	–	–	–	–	–	–	–
Yes	−0.096	−0.342	0.149	0.437	–	–	–	–	–	–
Diabetes mellitus										
No	1 (reference)	–	–	–	–	–	–	–	–	–
Yes	−0.098	−0.347	0.151	0.437	–	–	–	–	–	–
Coronary heart disease										
No	1 (reference)	–	–	–	–	–	–	–	–	–
Yes	0.036	−0.253	0.324	0.807	–	–	–	–	–	–
Dyslipidemia										
No	1 (reference)	–	–	–	–	–	–	–	–	–
Yes	−0.008	−0.248	0.232	0.950	–	–	–	–	–	–
Current smoking										
No	1 (reference)				1 (reference)					
Yes	−0.252	−0.462	−0.042	**0.019**	0.041	−0.047	0.129	0.359	0.466	2.146
Stroke										
No	1 (reference)	–	–	–	–	–	–	–	–	–
Yes	−12.195	−30.583	6.194	0.191	–	–	–	–	–	–
Antithrombotic therapy										
No	1 (reference)	–	–	–	–	–	–	–	–	–
Yes	−0.173	−0.499	0.153	0.295	–	–	–	–	–	–
*Clinical features*										
Baseline GCS score	−0.806	−1.168	−0.445	**<0.001**	0.039	−0.158	0.235	0.697	0.319	3.136
Baseline NIHSS score	0.754	0.516	0.992	**<0.001**	−0.026	−0.175	0.123	0.733	0.285	3.505
Onset to blood sampling	0.146	−0.130	0.422	0.294	–	–	–	–	–	–
Onset to CT scan	0.189	0.137	0.146	0.169	–	–	–	–	–	–
Baseline SBP	−0.694	−1.888	0.500	0.251	–	–	–	–	–	–
Baseline DBP	−0.573	−1.846	0.701	0.374	–	–	–	–	–	–
*Laboratory test results on admission*										
White blood cells	0.266	−0.040	1.017	**0.070**	−0.189	−0.368	−0.010	**0.039**	0.694	1.440
Blood glucose	0.733	0.055	1.410	**0.034**	−0.043	−0.282	0.196	0.723	0.651	1.537
Low‐density lipoprotein	0.082	−0.604	0.769	0.812	–	–	–	–	–	–
Plaque	−0.091	−0.738	0.556	0.781	–	–	–	–	–	–
APTT	−1.394	−2.743	−0.046	**0.043**	−0.160	−0.594	0.273	0.464	0.782	1.280
Fibrinogen	−0.298	−1.392	0.796	0.590	–	–	–	–	–	–
Hematoma volume	0.915	0.835	0.994	**<0.001**	0.870	0.793	0.946	**<0.001**	0.577	1.734
Serum occludin levels	0.425	0.252	0.598	**<0.001**	0.285	0.220	0.350	**<0.001**	0.699	1.431

*Note*: All continuous data were converted to lg to meet statistical requirements.

Abbreviations: APTT, activated partial thromboplastin time; DBP, diastolic blood pressure; GCS, Glasgow Coma Scale; ICH, intracranial hemorrhage; NIHSS, National Institutes of Health Stroke Scale; PHE, perihematomal edema volume; SBP, systolic blood pressure.

Potential variables (p〈0.1, bold in the 5th column) from simple line regression were further analyzed using multiple line regression to determine the variables (p〈0.05, bold in the 9th column), which are independently associated with PHE volumes.

In summary, these results suggested that the serum occludin level might be a biomarker reflecting PHE volumes in acute ICH patients.

## 4 DISCUSSION

This is the first study to report the association between baseline levels of serum occludin and PHE volumes inICH patients. The present study showed that ICH patients had a higher level of serum occludin than healthy controls. Patients with high levels of serum occludin at admission were more likely to have a severe PHE. The levels of serum occludin showed linear correlations with PHE volumes after adjustment of other factors from ICH patients. Taken together, these results suggested that serum occludin level was a potential biomarker reflecting the PHE volume in acute ICH patients.

With severe secondary damage after ICH, larger PHE volumes have been shown to be associated with poor clinic outcomes (modified Rankin Scale ≥3 or death).^22^, ^23^ Recent studies have discovered several clinical factors associated with the development of PHE, such as hematoma volume, hypertension history, and cerebral perfusion abnormalities.^24^, ^25^, ^26^ However, currently there is no blood biomarker indicating changes inPHE volumes, which could dynamically and frequently evaluate PHE volumes during the short period of acute ICH.

The molecular pathophysiology of PHE formation is complicated. Studies have shown that BBB disruption in brain tissues around hematomas was closely related to PHE formation after the onset of ICH.^27^, ^28^ Multiple mechanisms have been reported to be involved in BBB disruption‐induced PHE, such as inflammatory mediators, erythrocyte lysis, and activation of matrix metalloproteinases (MMPs).^29^, ^30^

Animal model studies of ICH have shown that increased MMP‐9 activation led to BBB disruption and brain edema.^31^, ^32^ Occludin, as a direct substrate of MMPs, was degraded into fragments from microvascular tissue by activated MMPs during cerebral injury.^33^ Our previous study showed that blood occludin levels correlated with the extent of BBB damage after ischemia stroke.^14^ Moreover, studies of an ICH animal model showed that hypoxia/ischemia occurred in brain tissues around the hematoma as a result of hematoma compression and focal inflammation.^34^ We therefore hypothesized that occludin in blood may reflect the PHE volume after the onset of ICH.

In the present study, we showed that the level of serum occludin at admission was higher in ICH patients with severe PHE, compared with those with mild PHE. Moreover, the levels of serum occludin were linearly correlated with PHE volumes in ICH patients and were independently associated with PHE volumes after adjusting for other clinical factors. Together, these findings suggested that the level of serum occludin may be a biomarker, indicating PHE volumes in early ICH patients.

Subtentorial ICH shows many differences from supratentorial ICH. Thesupratentorial ICH is more common in clinic and mainly caused by damages to the anterior circulation vessels. On the contrary, subtentorial ICHusually occurs due to damages to the posterior circulation vessels, leading to different clinical symptoms from supratentorial ICH. Therefore, to increase the homogeneity for analysis, subtentorial ICH was excludedfrom the present study. We will investigate whether serum occludin levels are associated with PHE in subtentorial ICH patients in further studies.

Detecting serum occludin levels has several advantages in reflecting PHE volumes, when compared with CT/MRI scans or other approaches during emergency examinations in clinical practice. First, the measurement of serum occludin is easier to conduct and can be conducted more frequently than CT/MRI scans, to screen potential deterioration in patients or to adjust treatment strategies by evaluating the severities of PHE in ICH patients during emergencies. Second, serum occludin detection, which was linearly correlated with PHE volumes, may be a more accurate indication of PHE development than other methods,^35^, ^36^ such as neurological examinations, intracranial pressure assessments, or cerebral blood flow assessments, which are related, but are not direct indicators of PHE volumes.

There are currently no effective drug targeting PHE‐induced severe secondary damages following ICH. Hyperosmolar agents, such as mannitol, have been frequently used in the clinic to reduce intracranial pressure. However, the effects of mannitol in reducing PHE or improving outcome in ICH patients are controversial and lack sufficient clinical randomized controlled efficacy trials.^37^, ^38^ Other potential methods (therapeutic hypothermia, intensive blood pressure reduction, and statin therapy) have not adequately shown that they prevented PHE formation or led to a good prognosis.^39^, ^40^, ^41^, ^42^ However, the results of the present study showed a high correlation between PHE volumes and serum occludin levels, which in the future may provide a novel target for alleviating PHE after ICH.

### 4.1 Limitations of the present study

First, this is a single‐center study to report the association between baseline levels of serum occludin and PHE volumes inICH patients. Large‐scale multicenter trials are warranted in the future to test the associations of serum occludin levels and PHE volumes. Second, in the present study we measured the association of serum occludin levels and PHE volumes within 24 h since stroke onset. It is crucial for understanding the development of PHE and serum occludin levels over 24 h in further studies. Third, the present study did not investigate whether serum occludin levels were associated with the prognosis of ICH. Further studies will focus on the association between serum occludin levels and prognosis of ICH patients. Fourth, in the present study, semiautomatic software was used to evaluate the volume of PHE based on CT scans. We consider applyingautomatic and objective‐imaging software in the future to improve the accuracy and objectivity of analysis. Finally, it was unclear how BBB disruption led to PHE and which molecules were involved in the mechanism of BBB disruption leading to PHE.

## 5 CONCLUSION

The present study showed that serum occludin levels at admission were independently correlated with PHE volumes in ICH patients, which may provide a biomarker indicating PHE volume change.

## CONFLICT OF INTEREST STATEMENT

There are no conflicts among the authors.

## Data Availability

The data that support the findings of this study are available from the corresponding author upon reasonable request.
